# Pathogens detected in ticks (*Ixodes ricinus)* feeding on red squirrels (*Sciurus vulgaris*) from city parks in Warsaw

**DOI:** 10.1007/s10493-024-00955-y

**Published:** 2024-09-09

**Authors:** Dorota Dwużnik-Szarek, Agata Beliniak, Wiktoria Malaszewicz, Dagny Krauze-Gryz, Jakub Gryz, Karolina D. Jasińska, Dagmara Wężyk, Anna Bajer

**Affiliations:** 1 Department of Eco-Epidemiology of Parasitic Diseases, Institute of Developmental Biology and Biomedical Sciences, Faculty of Biology, University of Warsaw, Miecznikowa 1, Warsaw, 02-096 Poland; 2 Department of Forest Zoology and Wildlife Management, Warsaw University of Life Sciences WULS-SGGW, Nowoursynowska 159, Warsaw, 02-776 Poland; 3 Department of Forest Ecology, Forest Research Institute, Sękocin Stary, Braci Leśnej 3, Raszyn, 05-090 Poland

**Keywords:** *Sciurus vulgaris*, *Ixodes ricinus*, *Borrelia*, *Rickettsia*, *Babesia*, Urban parks

## Abstract

The European red squirrel *(Sciurus vulgaris*) is a common host for *Ixodes ricinus* ticks in urban and rural habitats, however, studies on ticks and tick-borne pathogens (TBPs) of squirrels have not been conducted in Poland yet. Thus, the aims of the current study were to assess and compare the prevalence and abundance of ticks on red squirrels trapped at two sites in the Warsaw area (in an urban forest reserve and an urban park) and using molecular tools, to assess the genetic diversity of three pathogens (*Borrelia burgdorferi* sensu lato, *Rickettsia* and *Babesia* spp.) in *I. ricinus* ticks collected from squirrels. For the detection of *Rickettsia* spp. a 750 bp long fragment of the citrate synthase *gltA* gene was amplified; for *B. burgdorferi* s.l. 132f/905r and 220f/824r primers were used to amplify the bacterial *flaB* gene fragments (774 and 605 bp, respectively) and for *Babesia* spp., a 550 bpfragment of 18S rRNA gene was amplified. In total, 91 red squirrels were examined for ticks. There were differences in tick prevalence and mean abundance of infestation in squirrels from the urban forest reserve and urban park. Three species of *B. burgdorferi* s.l., *Rickettsia* spp., and *Babesia microti* were detected in ticks removed from the squirrels. Our results broaden knowledge of *S. vulgaris* as an important host for immature *I. ricinus* stages and support the hypothesis that red squirrels act as a reservoir of *B. burgdorferi*. Moreover, we conclude that red squirrels may also play a role in facilitating the circulation of other pathogens causing serious risk of tick-borne diseases in natural and urban areas.

## Introduction

Ticks and tick-borne pathogens (TBPs) constitute serious medical and veterinary problems not only in the natural rural environment but also in urban areas (Rizzoli et al. 2014; Kowalec et al. 2017, 2019). *Ixodes ricinus* (the castor been tick, Linnaeus, 1758) is the most common tick species occurring in Europe with important consequences for human health (Medlock et al. 2013; Cayol et al. 2017). The mean density of *I. ricinus* in urban public green areas in Europe can reach 6.9 ticks/100 m² or up to 13.2 ± 0.8/100 m^2^ adult plus nymphs, to even 306 nymphs/100 m^2^ (Kazimírová et al. 2016; Kowalec et al. 2017; Hansford et al. 2022). *Ixodes ricinus* ticks have been collected in highly-populated metropolitan areas, including London, Hannover, Berlin, Lyon, Zagreb, Ostrava, Košice or Zurich (Dobson et al. 2011; Pangrácová et al. 2013; Venclíková et al. 2014; Oechslin et al. 2017; Vucelja et al. 2020; Hauck et al. 2020; Mathews-Martin et al. 2020; Rubel et al. 2022). The presence of this tick species has been documented also in numerous Polish cities (Stanczak et al. 2004; Cisak et al. 2006; Kiewra and Lonc 2010; Buczek et al. 2014; Król et al. 2016; Kubiak et al. 2019; Liberska et al. 2021). In urban parks of Warsaw (Royal Łazienki Museum, Kabacki and Bielański Forests) mean tick density has been recorded as exceeding 10.1 ± 0.9 ticks/100 m^2^ (Kowalec et al. 2017).

Lyme borreliosis (LB) is the most important TBD and is caused by bacteria from *Borrelia burgdorferi* s.l. complex (Burn et al. 2023). In Poland, the incidence of Lyme borreliosis varies between years, however, an increasing trend has been observed in recent years. According to data published by the National Institute of Public Health - National Institute of Hygiene, in 2023 the incidence rate was 66.9 cases per 100,000 inhabitants, in 2022 − 45.9 and in 2021 − 32.7. In 2022, LB constituted 99% of human cases of tick-borne diseases (TBDs) (data from the Website of the Ministry of Health and the National Health Fund, https://pacjent.gov.pl/aktualnosc/uwazaj-na-kleszcze), and the incidence of borreliosis in previous years ranged from 33.1 in 2013 to 56.0 in 2017, although the figure for 2020 was lower at 33.7 cases.

Warsaw, the capital of Poland, is located in the Mazowieckie Voivodeship, where the incidence of LB is one of the highest in the country. To date, eight species of *Borrelia burgdorferi* s.l. have been detected in *I. ricinus* in Poland, differing in pathogenicity and host specificity (Gryczyńska et al. 2016; Wodecka et al. 2016; Kowalec et al. 2017). Among these, *Bo. burgdorferi* sensu stricto, *Bo. garinii* and *Bo. afzelii* are the main causes of LB (Branda and Steere 2021). In Europe, the mean prevalence of *Borrelia* spp. in questing *I. ricinus* (adults plus nymphs) collected from green urban areas has been recorded as 17.3% (range: 3.1– 38.1%) (Hansford et al. 2022). However, higher prevalence values in *I. ricinus* (adults and nymphs) have also been recorded, for example 25.5% in Hannover, Germany, (Glass et al. 2023), 28.1% in Prague, Czechia (Richtrová et al. 2022) and 40% in urban parks in Bulgaria (Blazhev et al. 2022). In three locations in Warsaw (Royal Łazienki Museum, Bielański and Kabacki Forests) prevalence of *Bo. burgdorferi* s.l. in *I. ricinus* (adults and nymphs) has been recorded as approximately 11% (Kowalec et al. 2017).

The DNA of *Bo. miyamotoi*, which can cause tick-borne relapsing fever (TBRF), has been identified in *I. ricinus* collected in city parks in Warsaw and other cities in Poland (Kowalec et al. 2017; Kubiak et al. 2019). The castor bean tick is known also to be a vector of the spotted fever group rickettsiae (SFG), bacteria of the *Rickettsia* genus (Kowalec et al. 2019; Welc-Falęciak et al. 2014) and of species of *Babesia* (*Ba. microti*, *Ba. divergens*, *Ba. venatorum*) (Azagi et al. 2021; Bajer and Dwużnik-Szarek 2021), which can cause human babesiosis, a disease that is especially hazardous for the splenectomized, elderly or immunocompromised individuals and pregnant women (Hildebrandt et al. 2021; Bajer et al. 2022).

Small rodents play an important role as hosts of *I. ricinus* larvae and nymphs (high intensity of *I. ricinus* infestation) and may act as reservoirs of TBPs (i.a. *Bo. miyamotoi*,* Bo. burgdorferi* s.l., tick-borne encephalitis virus (*Orthoflavivirus encephalitidis*), *Bartonella* spp. *Ba. microti*,* Neoehrlichia mikurensis*; Mihalca and Sandor, 2013; Grzybek et al. 2018; Dwużnik et al. 2019; Galfsky et al. 2019; Dwużnik-Szarek et al. 2021; Postler et al. 2023; Tołkacz et al. 2023). However, the rodent fauna of city parks is often depauperized in species (Gortat et al. 2014), and thus, red squirrels whose populations can reach very high densities in urban habitats (Beliniak et al. 2022), may act as important hosts for *I. ricinus* in urban areas (Sormunen et al. 2023), including Warsaw.

Pursuant to the Regulation of the Polish Ministry of the Environment (Dziennik Ustaw 2016 item 2183) released on 16th December 2016, red squirrels are partially protected species in Poland. They are a synurbic species, adapting well to environmental changes, occurring not only in natural forests but also in urban green areas (Krauze-Gryz et al. 2016; Babińska-Werka and Żółw 2012; Fey et al. 2016; Hämäläinen et al. 2018; Lurz et al. 2017; Fingland et al. 2021). The mean density of *S. vulgaris* can be higher in urban areas, compared to natural habitats, because of better access to supplemental food (walnuts, hazelnuts, peanuts) and other anthropogenic food sources (Krauze-Gryz et al. 2021; Fingland et al. 2021). Red squirrels are very common in green areas of Warsaw, especially in the park of the Royal Łazienki Museum (Babińska-Werka and Żółw 2008; Krauze-Gryz et al. 2020; Beliniak et al. 2022).

Data on tick infestation in red squirrels and on the pathogens carried by ticks from squirrels are limited (Humair and Gern 1998; Luu et al. 2021). However, some species of spirochaetes, including *Bo. garinii*, *Bo. afzelii*,* Bo. burgdorferi* s.s., and even *Bo. miyamotoi* have been recorded in tissue samples from red squirrels (skin, ear tissue samples; Humair and Gern 1998; Paulauskas et al. 2008; Pisanu et al. 2014). Furthermore, the DNA of other important TBPs, *Anaplasma phagocytophilum* (Ruyts et al. 2017) and *Hepatozoon* sp. (Modrý et al. 2021) has also been found in ticks collected from squirrels. However, to date no studies of tick infestations on red squirrels or of pathogens carried by squirrel-borne ticks have been conducted in Poland. We hypothesise that the large population of squirrels in urban parks and suburban forests contributes to the maintenance of high tick densities in these areas and, consequently, TBPs in the human environment. In urban areas, squirrels could significantly increase the transmission risk of TBPs (especially from the *Borrelia* genus) to humans and domestic animals (Pisanu et al. 2014; Ruyts et al. 2017).

## Materials and methods

### Study area and tick collection

Red squirrels were live-trapped at two sites within the administrative borders of Warsaw, the capital city of Poland.

#### An urban park: the Royal Łazienki Museum

**Fig. 1:**
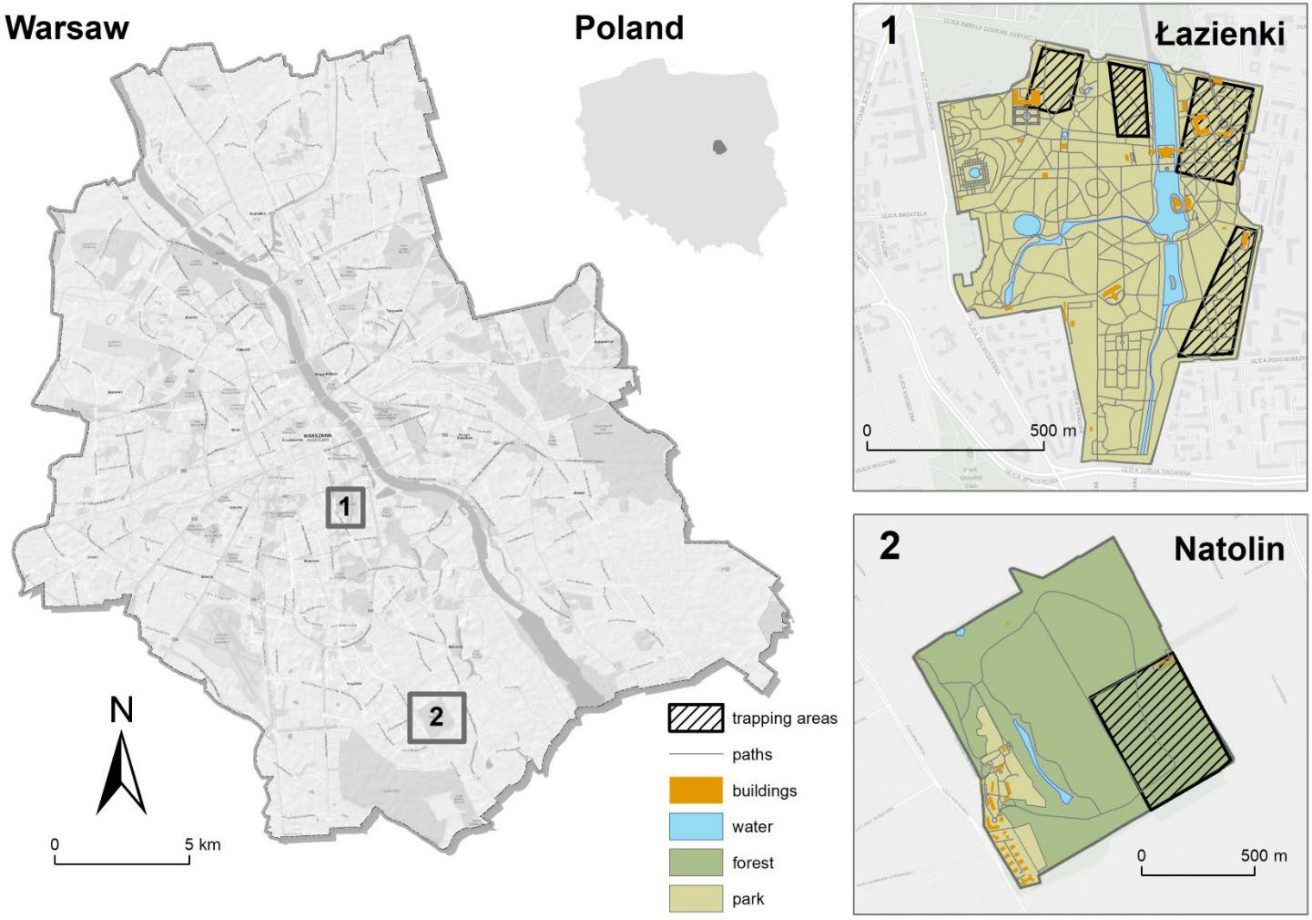
Study areas: (1) the Royal Łazienki Museum and (2) the Natolin Forest Reserve, where red squirrels were live-trapped (from: Body condition and breeding of urban red squirrels: comparison of two Populations affected by different levels of urbanization; Beliniak et al. 2022)

**Fig. 2:**
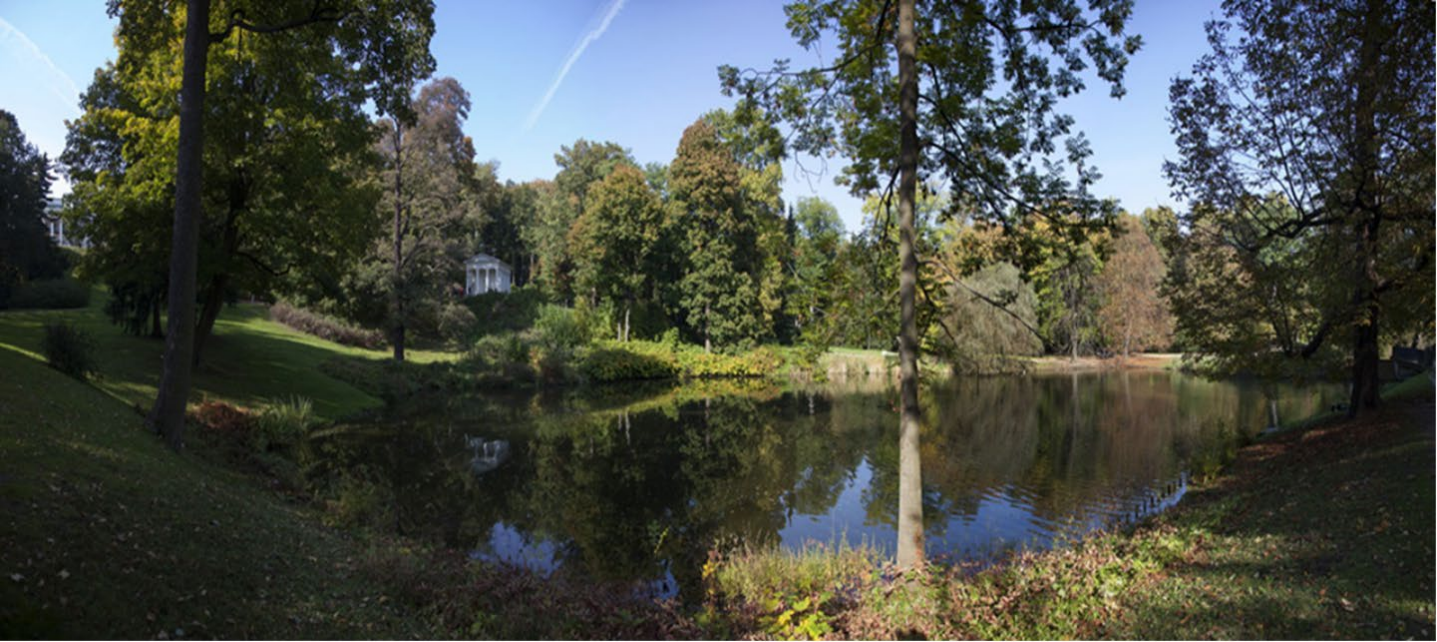
The Royal Łazienki Museum (from https://www.lazienki-krolewskie.pl)

The Royal Łazienki Museum is a historic park created by King Stanislaw II August Poniatowski in the 18th century. The park area (approximately 76 ha) is fenced (Kowalec et al. 2017) and located on the Vistula escarpment close to the city centre of Warsaw (52°12′53″N, 21°01′58″E; Fig. 1). According to an annual report, the number of tourists visiting the Royal Łazienki Museum has been increasing annually, from 3.5 million people in 2018 to more than 4 million visitors in 2022 (the Warsaw Tourism Organization, https://wot.waw.pl/Wiedza/). The park is a well-managed area, with numerous species of deciduous and coniferous trees, i.e. red oak (*Quercus rubra*), pedunculate oak (*Q. robur*), common hornbeam (*Carpinus betulus*), Norway spruce (*Picea abies*), silver fir (*Abies alba*), European larch (*Larix deciudua*) and open mowed areas (https://www.lazienki-krolewskie.pl/pl/ogrody/fauna-i-flora) (Fig. 2). Despite the lack of large animals, the presence of different potential hosts for *I. ricinus* has been observed, i.e. yellow necked mouse (*A. flavicollis*), striped field mouse (*A. agrarius*), the European hedgehog (*Erinaceus roumanicus*), the red fox (*Vulpes vulpes*) and passerine birds, especially black birds *Turdus merula* (Krauze-Gryz et al. 2016; Gryczyńska and Kowalec 2019; Dwużnik et al. 2020; Dwużnik-Szarek et al. 2021; https://www.lazienki-krolewskie.pl/pl/ogrody/fauna-i-flora). Companion animals are not allowed in the park.

#### An urban forest reserve: the Natolin Forest Reserve

The Natolin Forest Reserve is a remnant of the former natural Masovian forest. It is located about 10 km south of the Warsaw city centre (52°8′20″ N, 21°4′25″ E; Fig. 1), covering an area of 105 ha. The forest has been under protection since 1991 according to the Ordinance of the Minister of Environmental Protection, Natural Resources and Forestry, (M.P. 1991 No 38, item 273) and access to the park is limited (http://warszawa.rdos.gov.pl/las-natolinski-jest-udostepniony-spoleczenstwu-3). Special permission is required to enter the reserve. There are no paths/trails within the Natolin Forest Reserve. The tree stand consists of pedunculate oak, hornbeam, ash (*Fraxinus excelsior*), elms (*Ulmus* spp.), hazel (*Corylus* sp.), and black alder (*Alnus glutinosa*). It is a breeding place for foxes, badgers (*Meles meles*), and raccoon dogs (*Nyctereutes procyonoides*). Weasels (*Mustela nivalis*), European polecats (*M. putorius*), voles (*Microtus* spp.), common shrews *(Sorex araneus)*, Eurasian pygmy shrews (*S. minutus*), brown hares (*Lepus europaeus*), roe deer (*Capreolus capreolus*), beech martens (*Martes foina*) and hedgehogs have been observed (https://iwaw.pl/obiekt.php?p=312805017; Krauze-Gryz D., pers. obs; Jackowiak et al. 2021).

#### Red squirrel trapping methods

**Fig. 3:**
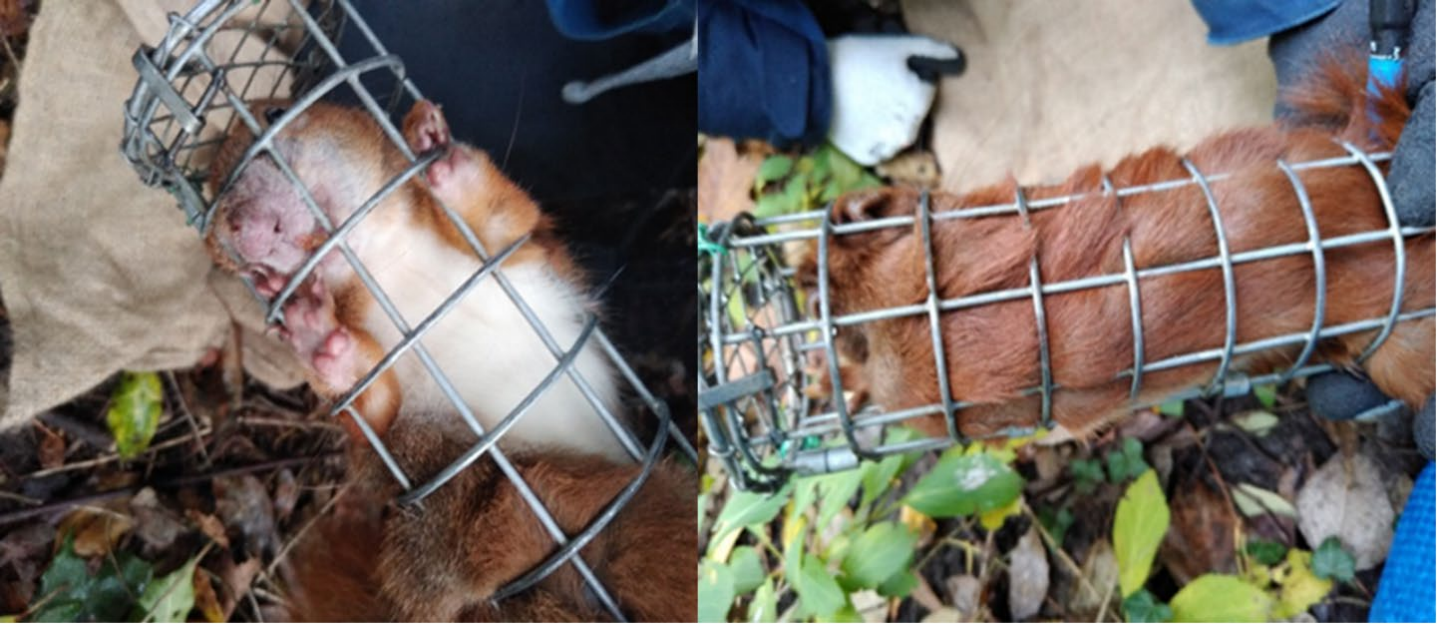
Red squirrel in the cone during examination (author DDS)

The methods utilised for trapping red squirrels have been fully described by Beliniak et al. (2021). In short, squirrels were live-trapped with 40 traps in the Natolin Forest Reserve and 30 traps in the Royal Łazienki Museum. Standard wire mesh live traps (51 × 15 × 15 cm) (manufactured by “Jerzyk” Jerzy Chilecki, Białowieża, Poland) were used. The traps were partly covered by dark plastic to provide shelter from rain and snow and were located on the ground or trees on a wooden platform. Live traps were pre-baited with hazelnuts and English walnuts for seven days. Then we trapped squirrels for four (in most cases) to nine days. The traps were baited and set in the morning (around 6–7 a.m., depending on the time of dawn), checked after 2–4 h, and secured for the night in a manner that prevented them from being closed. Every trapped squirrel was flushed into a wire mesh handling cone (Lurz et al. 1997; Fig. 3) to minimize stress during handling. Each newly trapped squirrel was individually marked with a numbered ear-tag 2 × 8 mm (National Tag&Band, Newport, KY, USA). Sex and age of trapped animals were recorded (Santicchia et al. 2018). Ticks were collected from handled animals and placed in 1.5 ml Eppendorf tubes, then transported to the laboratory of the Department of Eco-Epidemiology of Parasitic Diseases, Faculty of Biology, University of Warsaw, and frozen at -20ºC. Identification of collected ticks to species and developmental stage levels was facilitated by a morphological key (Estrada-Peña et al. 2018) and the use of a stereoscopic microscope Zeiss Stemi 508.

#### DNA extraction

The commercial Genomic Mini AX Tissue Spin kit (A&A Biotechnology, Gdańsk, Poland), was used for DNA extraction according to the manufacturer’s instructions. To increase the efficiency of the molecular component, larvae were processed in pools, comprising 2–10 larvae from a single host, while adult ticks and nymphs were processed individually.

In order to detect infection with *Bo. burgdorferi* s.l. and/or *Bo. miyamotoi*, nested PCR was performed using two sets of primers: 132f (5’-TGGTATGGGAGTTTCTGG-3’)/905r (5’-TCTGTCATTGTAGCATCTTT-3’) for amplification of 774 bp and 220f (5’CAGACAACAGAGGGAAAT-3’)/824r (5’-TCAAGTCTATTTTGGAAAGCACC-3’) for amplification of 605 bp product for the flagellin (*flaB*) gene (Wodecka et al. 2009). Reaction conditions were as provided by Kowalec et al. (2017) and Dwużnik-Szarek et al. (2021). For bacteria of the genus *Rickettsia*, primers CS409 (5’-CCTATGGCTATTATGCTTGC-3’) and Rp1258 (5’-ATTGCAAAAAGTACAGTGAACA-3’) were used, amplifying a 750 bp fragment of the citrate synthase (*gltA*) gene (Roux et al. 1997) with modified reaction conditions as described by Kowalec et al. (2019).

Nested PCR was performed to detect *Babesia* DNA. In the first reaction, primers CryptoF (5’AACCTGGTTGATCCTGCCAGT3’)/ CryptoR (5’GCTTGATCCTTCTGCAGGTTCACCTAC3’) were used to amplify about 1200 bp gene fragment of small subunit ribosomal RNA (18S rRNA gene). In the second reaction, primers BabGR2 (5’-CCAAAGACTTTGATTTCTCTC-3’)/ BabGF2 (5’-GYYTTGTAATTGGAATGATGG-3’) were used to amplify a 550 bp fragment (Bonnet et al. 2007; Tołkacz et al. 2017). The reaction conditions were the same for both PCR steps and described in details by Tołkacz et al.(2017).

Selected PCR products were commercially sequenced by Genomed company (Warsaw, Poland). Sequence alignments and analyses were carried out using BLAST-NCBI and MEGA X software (Kumar et al. 2018). Phylogenetic analyses were performed using the Maximum Likelihood method of tree construction. The evolutionary model was chosen in accordance with the data (following implemented model test in MEGA X) and bootstrapped over 1000 randomly generated sample trees (Dwużnik-Szarek et al. 2021). For *Borrelia* spp., the evolutionary history was inferred by using the Maximum Likelihood method and Hasegawa-Kishino-Yano + G model) based on the approximately 605 bp fragment of *fla*B gene, incorporating seven sequences obtained in this study and 18 reference sequences from GenBank. For *Rickettsia* spp. analyses, the evolutionary history was inferred by using the Maximum Likelihood method a Hasegawa-Kishino-Yano + G model based on the approximately 750 bp fragment of *gltA* gene The analysis included 22 nucleotide sequences (13 obtained in this study and 9 sequences from GenBank).

### Statistical analyses

Statistical analyses were conducted for evaluation of the effect of host intrinsic factors (sex and age), and extrinsic factors including study site, year and month of trapping on tick infestation and presence of pathogen DNA in ticks collected from squirrels.

All statistical analyses were conducted in PS IMAGO PRO Academic v.7 (IBM SPSS Statistics software, institutional license purchased by the University of Warsaw, Warsaw, Poland). The statistical approach analysis has been documented in our previous papers (Behnke et al. 2001; Mierzejewska et al. 2020; Dwużnik-Szarek et al. 2021).

For analysis of prevalence (% positive-tested samples), we applied maximum likelihood techniques based on log-linear analysis of contingency tables in IBM SPSS software. SITE of red squirrel trapping (two levels: an urban forest reserve and an urban park), SEX of squirrels (males and females), AGE (two levels: young, adult), YEAR (two levels: 2019 and 2020), MONTH (five levels: March, May, June, July, September), were used as the factors in models with the presence or absence of ticks and referred to as INFESTATION or PRESENCE/ABSENCE of pathogen DNA considered as a binary factor (0, 1). For each level of analysis in turn, beginning with the most complex model, involving all possible main effects and interactions, those combinations that did not contribute significantly to explaining variation in the data were eliminated in a stepwise fashion beginning with the highest-level interaction (backward selection procedure). A minimum sufficient model was then obtained, for which the likelihood ratio of χ^2^ was not significant, indicating that the model was sufficient in explaining the data. The importance of each term in interactions involving INFESTATION or PRESENCE/ABSENCE of pathogen in the final model was assessed by the probability that its exclusion would alter the model significantly and these values are given in the text, assessed by a likelihood ratio test between nested models with and without each factor of interest.

General linear models (GLMs; analyses also conducted in IBM SPSS software) were used for the analysis of mean intensity of tick infestation on squirrels, using models with normal errors, incorporating SEX and AGE of trapped squirrels, YEAR, SITE and MONTH in which the squirrels were trapped as fixed factors. Means are presented with the standard error of the mean (S.E.).

### Ethical approval

The study was performed with the approval of the Regional Director for Environmental Protection (license number: WPN-I.6205.124.2018.AS and WPN-I.6401.208.2018.PF). Trapping and handling squirrels complied with current regulations on conducting experimental studies on animals in Poland and Europe and were carried out under a license obtained from the Second Local Ethical Committee in Warsaw (license number WAW2/072/2018).

## Results

In total, 91 (52 males and 39 females; 12 young and 79 adults) red squirrel individuals were examined for ticks (some were re-trapped, resulting in 185 checks), including records of 105 males and 80 females; 173 records of adults and 12 records of juvenile individuals (Table 1). Sixty red squirrels were from the urban forest reserve and 31 from the urban park. In total, 458 *I. ricinus* ticks (72 larvae, 372 nymphs, 12 females, and 2 males) were collected (Table 2A).

**Table 1:** Number of (re)trapped red squirrels recorded by year, site, month, sex and age

Year	Site	Month	Sex		Age	
			Male	Female	Young	Adult
2019	Royal Łazienki Museum	March	0	0	0	0
		May	9	10	4	15
		June	2	1	0	3
		July	12	6	4	14
		September	5	13	1	17
		Total:	28	30	9	49
	Natolin Forest Reserve	March	0	0	0	0
		May	7	5	1	11
		June	0	0	0	0
		July	9	3	0	12
		September	6	5	1	10
		Total	22	13	2	33
		Subtotal (Royal Łazienki Museum + Natolin Forest Reserve)	50	43	11	82
2020	Royal Łazienki Museum	March	17	15	1	31
		May	8	2	0	10
		June	0	0	0	0
		July	0	6	0	6
		September	0	0	0	0
		Total	25	23	1	47
	Natolin Forest Reserve	March	8	3	0	11
		May	1	0	0	1
		June	11	4	0	15
		July	10	7	0	17
		September	0	0	0	0
		Total	30	14	0	44
		Subtotal (Royal Łazienki Museum + Natolin Forest Reserve)	55	37	1	91
		Total all 2019 + 2020	105	80	12	173

**Table 2:** Number of ticks (A) total tick collected from red squirrels; (B) examined by molecular methods

(A) Year	Site	Month	Life stage			
			larvae	nymph	male	female
2019	Royal Łazienki Museum	March	0	0	0	0
		May	5	19	1	4
		June	0	1	0	0
		July	0	0	0	1
		September	0	0	0	0
		Total:	5	20	1	5
	Natolin Forest Reserve	March	0	0	0	0
		May	9	22	0	0
		June	0	0	0	0
		July	4	7	0	0
		September	8	54	0	0
		Total:	21	83	0	0
		Total (Royal Łazienki Museum + Natolin Forest Reserve)	26	103	1	5
2020	Royal Łazienki Museum	March	0	8	1	3
		May	8	39	0	1
		June	0	0	0	0
		July	0	3	0	2
		September	0	0	0	0
		Total:	8	50	3	6
	Natolin Forest Reserve	March	0	28	0	0
		May	1	16	0	0
		June	33	129	0	0
		July	4	46	0	1
		September	0	0	0	0
		Total:	38	219	0	1
		Total (Royal Łazienki Museum + Natolin Forest Reserve)	46	269	3	7
		Total all (2019 + 2020)	72	372	2	12
(B) Year	Site	Month	Life stage			
			larvae	nymph	male	female
2019	Royal Łazienki Museum	March	0	0	0	0
		May	5	19	1	4
		June	0	0	0	0
		July	0	0	0	1
		September	0	0	0	0
		Total:	5	19	1	5
	Natolin Forest Reserve	March	0	0	0	0
		May	8	22	0	0
		June	0	0	0	0
		July	4	7	0	0
		September	8	54	0	0
		Total:	20	83	0	0
		Total (Royal Łazienki Museum + Natolin Forest Reserve)	25	102	1	5
2020	Royal Łazienki Museum	March	0	2	0	0
		May	8	37	0	1
		June	0	0	0	0
		July	0	2	0	2
		September	0	0	0	0
		Total:	8	41	0	3
	Natolin Forest Reserve	March	0	14	0	0
		May	0	11	0	0
		June	26	93	0	0
		July	2	45	0	1
		September	0	0	0	0
		Total:	28	163	0	1
		Total (Royal Łazienki Museum + Natolin Forest Reserve)	36	205	0	4
		Total all (2019 + 2020)	61	306	1	9

### Prevalence of tick infestation

The general infestation rate was 56.8% [95% CI: 49.56–63.74]. In 2019, the prevalence of tick infestation was lower than in 2020 (48.4% [95%CI: 38.41–58.46] vs. 65.2% [95%CI: 55.13–74.36]) (tick presence/absence × YEAR: χ^2^_1_ = 5.37, *P* < 0.02). Significantly higher tick infestation was observed in May and June in comparison to the remaining months (tick presence/absence × MONTH: χ^2^_4_ = 27.36, *P* < 0.001; Table 3).

**Table 3:** Prevalence, CI and mean intensity of tick infestation of red squirrels trapped in Warsaw (urban park + urban forest reserve) by month ± S.E

Month	March	May	June	July	September
Prevalence (%)	39.5 (17/43)	78.6 (33/42)	88.9 (16/18)	52.8 (28/53)	37.9 (11/29)
95% CI	25.99–54.44	64.53–88.83	68.88–97.67	39.54–65.82	22.09–56.05
Mean intensity of infestation ± S.E.	2.35 ± 0.51	3.79 ± 0.66	10.19 ± 1.96	2.43 ± 0.43	5.64 ± 1.34

The prevalence of *I. ricinus* infestation among squirrels trapped in the urban forest reserve (87.3%), was twice higherin the urban park (34%) (tick presence/absence × SITE: χ^2^_1_ = 57.22, *P* < 0.001). Similar tick prevalence was observed for male and female red squirrels (60.4% vs. 51.4%; (*P* > 0.05), and there was no significant difference in prevalence between age classes as well (*P* > 0.05).

### Mean intensity of tick infestation

Overall, the mean intensity of tick infestation was 4.19 ± 0.48 ticks/individual. As with prevalence, significantly higher mean intensity was observed in animals trapped in 2020 (5.38 ± 0.76 ticks/individual) in comparison to 2019 (3.00 ± 0.44 ticks/individual; main effect of YEAR on *I. ricinus* intensity of infestation: *F*_1,104_ = 6.28, *P* = 0.01). Mean intensity was two times higher among squirrels from the urban forest reserve compared to those from the urban park (5.25 ± 0.67 and 2.67 ± 0.49 ticks/individual, respectively; main effect of SITE on *I. ricinus* intensity of infestation: *F*_1,104_ = 6.80, *P* = 0.01) and it was higher for red squirrels trapped in June and September compared to the remaining months (main effect of MONTH on *I. ricinus* intensity of infestation: *F*_4,104_ = 10.36, *P* < 0.001; Table 3).

### Molecular detection of pathogen DNA in ticks

In total, 377 *I. ricinus* ticks from 55 rodents were examined by molecular techniques, encompassing 9 females, one male, 306 nymphs, and 61 larvae in 34 pools (350 samples) (Table 2B).

The DNA of a single pathogen was identified in 15.9% [95% CI: 12.45–20.11]of ticks, and co-infection was detected only in one nymph from the urban park (*Ba. microti* + *Bo. afzelii*; prevalence of co-infection = 0.3%). A similar prevalence of pathogen DNA was observed in ticks collected in 2019 and 2020 (18%[95% CI: 12.07–25.3] and 14.9% [95% CI: 10.65–19.99], respectively; *P* > 0.05). There were also no differences in the prevalence of pathogens detected in ticks collected from squirrels trapped in different months. A higher prevalence of pathogen DNA was found in ticks collected from the urban forest reserve (18.3%)[95% CI: 14.13–23.31] compared to ticks from the urban park (7.5%; [95% CI: 3.27–15.19]; pathogen DNA presence/absence × SITE: *χ*^2^_1_ = 6.22, *P* = 0.01). Pathogen DNA was identified in 20% of larval pools, 14% of nymphs, and 10% of adults (*P* > 0.05).

### Detection of *Borrelia* spp. DNA in ticks from squirrels

Total prevalence of *Bo. burgdorferi* s.l. was 2.5% [95% CI:1.28–4.64] (9/350; one larva, eight nymphs and one female collected from nine rodents with two retrapped two times). Prevalence differed between years of tick collection and was 5.4% [95% CI: 2.48–10.44] in 2019 but only 0.9% [95%CI: 0.19–2.86] in 2020 (YEAR × *Borrelia* spp. presence/absence: *χ*^2^_1_ = 6.46, *P* = 0.01). *Borrelia* spp. DNA was detected only in ticks collected in May (6.3%) [95% CI: 2.85–12.3] and in September (3.3%)[95% CI: 0.69–10.1] (MONTH × *Borrelia* spp. presence/absence: χ^2^_4_ = 13.97, *P* = 0.01). There were no differences in prevalence between sites and tick stages (*P* > 0.05).

**Fig. 4:**
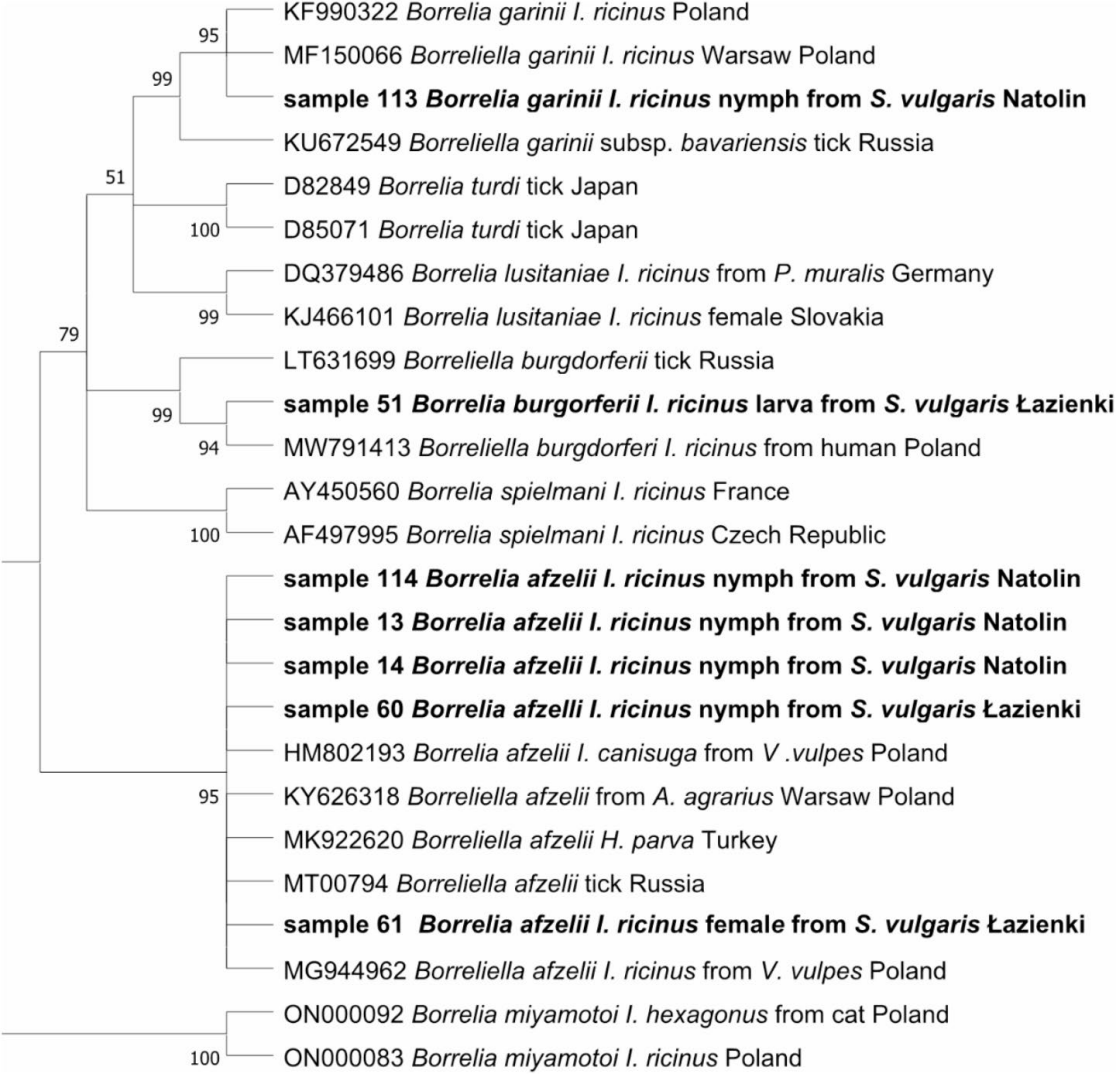
Molecular phylogenetic analysis of *flaB* gene fragment of *Borrelia burgdorferi* s.l. (605 bp). The evolutionary history was inferred by using the Maximum Likelihood method and Hasegawa-Kishino-Yano model. The tree with the highest log likelihood (-1557.49) is shown. The percentage of trees in which the associated taxa clustered together is shown next to the branches. Initial tree(s) for the heuristic search were obtained automatically by applying Neighbor-Join and BioNJ algorithms to a matrix of pairwise distances estimated using the Maximum Composite Likelihood (MCL) approach, and then selecting the topology with superior log likelihood value. A discrete Gamma distribution was used to model evolutionary rate differences among sites (5 categories (+ *G*, parameter = 0.4006)). This analysis involved 25 nucleotide sequences. There were a total of 472 positions in the final dataset. Evolutionary analyses were conducted in MEGA X

Seven sequences from nine positive samples of *Borrelia* spp. (four from nymphs from the urban forest reserve and one nymph, one female, and one larval pool from the urban park) were obtained. Three species were identified, *Bo. afzelii*,* Bo. garinii*, and *Bo. burgdorferi* s.s. Five *Bo. afzelii* sequences with a 99.9% similarity to *B. afzelii* from *I. ricinus*, Turkey (GenBank Acc. No. MK922620) were detected in nymphs from the urban forest reserve as well as in a nymph and a female from the urban park. One *Bo. garinii* (100% identity to *Bo. garinii* from *I. ricinus*, Poland (MK604263)) was found in a nymph from the urban forest, and one *Bo. burgdorferi* s.s. (99.8% identity to *Bo. burgdorferi* s.s. from *I. ricinus*, Poland (MW791413)) was detected in a larval pool from the urban park. The phylogenetic tree of *Borrelia* spp. is presented in Fig. 4.

### Detection of *Babesia* sp. DNA in ticks from squirrels

The DNA of *Babesia* sp. was found only in one nymph from a squirrel from the urban park (total prevalence 0.3% [95% CI: 0.03–1.33]), and it had 100% identity to *B. microti* from *I. ricinus* from Turkey (OM066130).

### Detection of *Rickettsia* spp. DNA in ticks from squirrels

Overall prevalence of *Rickettsia* spp. was 13.9% [95%CL: 10.67–17.93] 49/350; six pooled larvae, forty nymphs and and three females collected from 26 rodents with 12 of individuals were retrapped two to four times), and was similar in both years, 13.1% [95%CI: 8.24–19.96] in 2019 and 14.3% [95%CI: 10.27–19.48] in 2020, and in the months of trapping (*P* > 0.05). Prevalence of *Rickettsia* was three times higher among ticks from squirrels from the urban forest reserve than those from the urban park (16.5% [95%CI: 12.49–21.3] vs. 5.0%[95%CI: 1.76–11.73]) (SITE × *Rickettsia* spp. presence/absence: χ^2^_1_ = 8.22, *P* = 0.004). *Rickettsia* DNA was detected in 30% [95%CI: 9.27–60.58] of adult ticks, 17% [95%CI: 7.72–32.81] of nymphs and 13% of larvae pools [95%CI: 9.65–17.19] (*P* > 0.05).

**Fig. 5:**
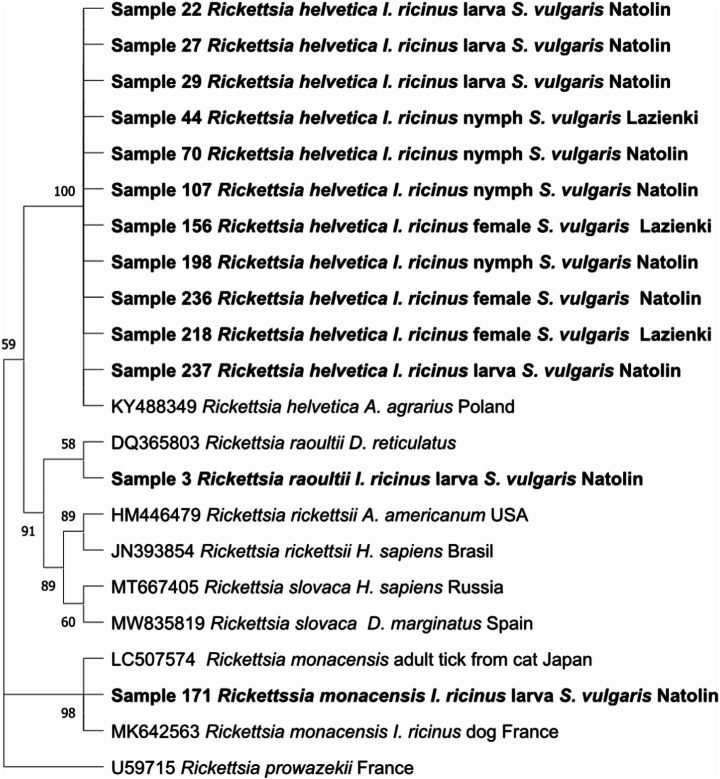
Molecular phylogenetic analysis of citrate synthase (*gltA*) gene fragment of *Rickettsia* (750 bp). The evolutionary history was inferred by using the Maximum Likelihood method and Hasegawa-Kishino-Yano model. The tree with the highest log likelihood (-1343,27) is shown. The percentage of trees in which the associated taxa clustered together is shown next to the branches. Initial tree(s) for the heuristic search were obtained automatically by applying Neighbor-Join and BioNJ algorithms to a matrix of pairwise distances estimated using the Maximum Composite Likelihood (MCL) approach, and then selecting the topology with superior log likelihood value. A discrete Gamma distribution was used to model evolutionary rate differences among sites (5 categories (+ *G*, parameter = 0,2041)). This analysis involved 22 nucleotide sequences. There were a total of 667 positions in the final dataset. Evolutionary analyses were conducted in MEGA X

Thirteen *Rickettsia* PCR-positive products were sequenced (from five larval pools, three nymphs, and one female from the urban forest reserve as well as from two nymphs and one female from the urban park). Three *Rickettsia* species were identified: *R. raoultii* from larval pool from the urban forest reserve showed the 100% identity to the sequence from *D. reticulatus* from France (GenBank DQ365803); eleven *R. helvetica* sequences (from two females and one nymph from the urban park as well as four larvae, three nymphs and one female from the urban forest reserve) were 100% identical with a sample from *A. agrarius* from Poland (KY488349); and one *R. monacensis* from larval pool from the urban forest reserve had the highest identity (99.81%) with the sequence from tick collected from cat in Japan (GenBank Acc. No. LC507574; Fig. 5).

## Discussion

In the present study, we confirmed the role of red squirrels as important hosts for *I. ricinus* ticks and recovered the DNA of seven pathogens from the ticks collected from these animals captured in the urban areas.

The prevalence of infestation of red squirrels by the castor bean tick was 56.8%. A much higher infestation by *I. ricinus* (88%) was observed in squirrels caught in Norway (Mysterud et al. 2021). This difference may be due to the higher number of animals tested in our study (91 squirrels in our study and 17 from Norway) and the longer period of sampling, including months of lower tick activity. High tick infestation of red squirrels has also been recorded on two islands, Brownsea (88.9%) and Arran (53.2%) in the UK (Luu et al. 2021). In similar studies conducted in France, the recorded prevalence of *I. ricinus* infestation on squirrels reached 34% (Romeo et al. 2013). However, even a lower infestation of *I. ricinus* (25%) was identified among road-killed red squirrels in the UK (Simpson et al. 2013). Examining a host under anaesthesia and/or live trapped animals as in our case, reduces the likelihood of postmortem loss of ectoparasites, and consequently, ectoparasite burden can be expected to be higher compared with road-killed animals (Tahir et al. 2020).

In our study, we trapped red squirrels in two urban settings. A significantly higher prevalence of *I. ricinus* infestation was noted among squirrels from the urban Natolin Forest Reserve compared to those from the Royal Łazienki Museum. The Royal Łazienki Museum is a managed park, located near the city centre, and the lawns are regularly mowed, which may reduce the prevalence of tick infestation in squirrels. It has been demonstrated previously that regular mowing and maintenance of green areas can significantly contribute to reducing tick densities (Medlock et al. 2012; Bajer et al. 2017), thus the number of infested hosts. In the study conducted by Kowalec et al. (2017), higher tick density (collected from vegetation) was recorded in the natural Białowieża Forest in comparison to three urban parks in Warsaw (Kabacki and Bielański forests and the Royal Łazienki Museum; Kowalec et al. 2017).

The overall prevalence of *Bo. burgdorferi* s.l. infection in *I. ricinus* collected from squirrels in our study was low (2.5%). More than five times higher prevalence of *Borrelia* spp. (12.8%) was found in ticks collected from red squirrels in the UK (Luu et al. 2021). The high prevalence of *Borrelia*-positive *I. ricinus* ticks collected from vegetation can be a source of infection for many host species, including red squirrels occurring in Warsaw parks. In 2009–2010, a prevalence of 5.7% for *Borrelia* spp. was observed in adult ticks collected from vegetation in the city park of the Royal Łazienki Museum (Chmielewski et al. 2011). During the study conducted in 2012–2015, the prevalence of *Borrelia*-positive *I. ricinus* (adults plus nymphs) reached more than 17% (Kowalec et al. 2017). Those studies show that infection of *Borrelia* spp. could fluctuate between years. Similar to the prevalence of *Babesia canis* in *Dermacentor reticulatus* tick populations, that may indicate the incidence of babesiosis in dogs, prevalence of *Borrelia* in *I. ricinus* collected from vegetation may indicate the risk of infection of potential hosts (Dwużnik-Szarek et al. 2021).

The sequencing of our samples revealed the presence of three genospecies of *Borrelia*: *Bo. afzelii*, *Bo. garinii*, and *Bo. burgdorferi* s.s. *Borrelia afzelii and Bo. burgdorferi* s.s. were detected in ticks collected from rodents trapped in Royal Łazienki Museum, while in Natolin Forest Reserve *Bo. garini* and *Bo. afzelii* were detected. All of *Borrelia* genospecies found in our study were previously noted in *I. ricinus* collected from vegetation in urban and natural areas (Ruyts et al. 2018; Heylen et al. 2019, Grochowska et al. 2020). Research conducted by Kowalec et al. (2017) revealed dominance of *B. afzelii* (69.3%) in urban and *B. garinii* (48.1%) in natural areas in ticks collected from vegetation (Kowalec et al. 2017). In Switzerland, two *Borrelia* species (*Bo. afzelii* and *Bo. burgdorferi* s.s) were identified in ticks collected from red squirrels (Humair and Gern 1998). In that study, 15 squirrel skin samples were also tested, in which both *Borrelia* species were detected (Humair and Gern 1998). Many other studies conducted in France, Belgium and Hungary have confirmed the presence of *Bo. afzelii*, *Bo. miyamotoi*, *Bo. garinii*, *Bo. burgdorferi* s.s., *Bo. bissetti*, and *Bo. carolinensis* in red squirrel tissues/blood samples with prevalence ranging from 3.5% to even 45% (Pisanu et al. 2014; Ruyts et al. 2017; Szekeres et al. 2019; Majerová et al. 2020). Interestingly, in our study, the sequences of *Bo. garinii* (from *I. ricinus* nymph collected from red squirrels trapped in the Natolin Forest Reserve; Fig. 4) clustered close with the sequence identified as *Bo. garinii* subsp. *bavariensis* (KU372549). Both genospecies differ in their suspected reservoir hosts (*B. garinii*: birds, *B. bavariensis*: rodents) and both have been detected in *I. ricinus* in Europe (Margos et al. 2019). *Borrelia bavariensis* (previously known as *Bo. garinii* OspA serotype 4) was raised to species level in 2009 and thus separated from its sister species *B. garinii*, however, using just one gene marker might not allow differentiation between them (Margos et al. 2019).

We detected only one *Ba. microti*-positive *I. ricinus* tick, a nymph from the red squirrel from the urban park. To the best of our knowledge, there are just two reports of *Babesia* presence in red squirrels and one finding of *Babesia* DNA in ticks feeding on squirrels (Tsuji et al. 2006; Lipatova et al. 2017). In Japan, a *B. microti*-like strain was found in three of six examined squirrels (Tsuji et al. 2006). Surprisingly, in red squirrels in Lithuania, the DNA of *Babesia* spp. was detected in 57.6% of squirrels and13.4% of ticks collected from these hosts (Lipatova et al. 2017). Our result (prevalence 0.3%) however, was similar to the very low prevalence of *Babesia* spp. (usually below 2%) recorded in *I. ricinus* ticks collected from vegetation in many countries in Europe, including Poland (Cotte´ et al. 2010; Schorn et al. 2011; Egyed et al. 2023; Øines et al. 2012; Sytykiewicz et al. 2015; Hamšíková et al. 2016; Karlsson and Andersson 2016; Wilhelmsson et al. 2021; Grochowska et al. 2022; Wondim et al. 2022). Interestingly, the *Ba. microti* positive nymph was co-infected with *Bo. afzelii.*

The role of the red squirrel as a reservoir of *Rickettsia* is poorly investigated. We found only one report, based on work conducted in Lithuania, where the DNA of *Rickettsia* spp. was found in red squirrel tissues (prevalence 12.1%) and in ticks collected from this host (prevalence about 23%; Lipatova et al. 2017). In our study, the overall prevalence of the *Rickettsia* spp. detected in ticks collected from red squirrels was the highest of all tested pathogens and reached almost 14%. The prevalence in the urban park was much lower (5%) than in the urban forest (16%). Higher rate of infection of ticks with bacteria of the *Rickettsia* genus may be caused by the higher prevalence and intensity of tick infestation observed in squirrels from the natural forest than from the urban park. Bacteria from the *Rickettsia* genus are usually prevalent in *I. ricinus* populations including ticks collected from vegetation in the Warsaw areas. Previous studies from the Royal Łazienki Museum revealed *Rickettsia* prevalence of 6.5-7.7% or even 26% in questing *I. ricinus* (Chmielewski et al. 2009; Welc-Falęciak et al. 2014; Kowalec et al. 2019), which is in line with our findings from this location.

In the present study, three species of *Rickettsia* were identified: *R. raoultii*, *R. helvetica*, and *R. monacensis*, all pathogenic to humans. Every year the National Institute of Public Health reports several cases of rickettsiosis in Poland (https://www.pzh.gov.pl/) but the number of cases seems to be underestimated due to the non-specific symptoms of the infection (Kirczuk et al. 2021). *Rickettsia monacensi*s was first identified in 1989 in Munich, Germany (Simser et al. 2002). In Poland, the first finding of *R. monacensis* in *I. ricinus* was published in 2012 (Rymaszewska and Piotrowski 2013). *Rickettsia raoultii* is highly prevalent in *D. reticulatus* populations (Dwużnik-Szarek et al. 2022) but has been also noted in *I. ricinus* (Stańczak et al. 2012). The presence of *R. raoultii* and *R. monacensis* DNA in *I. ricinus* larvae collected from red squirrels may indicate the source of these pathogens from the host. However, as bacteria from the genus *Rickettsia* can be transmitted via transstadial and transovarial routes, the infections could be inherited (Nováková and Šmajs 2018). Another possibility is via the meal contamination phenomenon described by Dwużnik et at. (2019). We cannot exclude that *Rickettsia-*positive larvae co-fed with other ticks infected with these pathogens on the same hosts. This is an interesting observation that confirms the value of conducting research on tick-host-pathogen interactions. *Rickettsia helvetica*, the most commonly detected species in our samples (in all life stages), has been detected frequently in questing *I. ricinus* in Poland, also from the area of Warsaw (Wodecka et al. 2014; Stańczak et al. 2012; Kowalec et al. 2019). All three *Rickettsia* species were detected in ticks from the urban forest, while in the urban park, only *R. helvetica* was found.

## Conclusions

Red squirrels constitute a permanent element of the urban fauna and are a significant host for *I. ricinus*, especially for larvae and nymphs. Considering the large populations of red squirrels in Warsaw, this rodent may act as an important reservoir of tick-borne pathogens, mainly bacteria of the *B. burgdorferi* s.l. complex. The detection of the DNA of *Babesia* and *Rickettsia* in ticks collected from squirrels indicates the persistence of these pathogens in the urban environment. It is now necessary to verify whether, as in the case of *Borrelia*, the red squirrel can be a reservoir of both *Babesia* and *Rickettsia* spp. and thus contribute to the circulation of these pathogens in Warsaw.

## Data Availability

We declare all data is being provided within this manuscript.
